# The curious case of peroxiredoxin-5: what its absence in aves can tell us and how it can be used

**DOI:** 10.1186/s12862-018-1135-z

**Published:** 2018-02-08

**Authors:** Marc Pirson, André Clippe, Bernard Knoops

**Affiliations:** Group of Animal Molecular and Cellular Biology, Institut des Sciences de la Vie (ISV), Université catholique de Louvain, 4-5 Place Croix du Sud, 1348 Louvain-la-Neuve, Belgium

**Keywords:** Peroxiredoxin-5, Aves, Birds, Amniotes, Vertebrates, TRMT112, Gene loss, Gain-of-function

## Abstract

**Background:**

Peroxiredoxins are ubiquitous thiol-dependent peroxidases that represent a major antioxidant defense in both prokaryotic cells and eukaryotic organisms. Among the six vertebrate peroxiredoxin isoforms, peroxiredoxin-5 (PRDX5) appears to be a particular peroxiredoxin, displaying a different catalytic mechanism, as well as a wider substrate specificity and subcellular distribution. In addition, several evolutionary peculiarities, such as loss of subcellular targeting in certain species, have been reported for this enzyme.

**Results:**

Western blotting analyses of 2-cys PRDXs (PRDX1–5) failed to identify the PRDX5 isoform in chicken tissue homogenates. Thereafter, via *in silico* analysis of PRDX5 orthologs, we went on to show that the *PRDX5* gene is conserved in all branches of the amniotes clade, with the exception of aves. Further investigation of bird genomic sequences and expressed tag sequences confirmed the disappearance of the gene, though *TRMT112*, a gene located closely to the 5′ extremity of the *PRDX5* gene, is conserved. Finally, using *in ovo* electroporation to overexpress the long and short forms of human PRDX5, we showed that, though the gene is lost in birds, subcellular targeting of human PRDX5 is conserved in the chick.

**Conclusions:**

Further adding to the distinctiveness of this enzyme, this study reports converging evidence supporting loss of *PRDX5* in aves. In-depth analysis revealed that this absence is proper to birds as *PRDX5* appears to be conserved in non-avian amniotes. Finally, taking advantage of the *in ovo* electroporation technique, we validate the subcellular targeting of human PRDX5 in the chick embryo and bring forward this gain-of-function model as a potent way to study PRDX5 functions in vivo.

## Background

Reactive oxygen and nitrogen species (ROS/RNS) are toxic compounds generated out of the cell, but also intracellularly through the electron transport chain, and in various physiological and pathophysiological situations. Their toxicity arises from unpaired electrons, or the oxygen and nitrogen content within these molecules that render them highly reactive towards biological components such as DNA, lipids and proteins [1]. However, at moderate concentrations ROS/RNS have also been shown to be implicated in numerous cellular processes, including cell signaling [2, 3]. Due to this dual role, fine control of ROS/RNS concentrations at the cellular level is essential, a function performed by the different enzymatic and non-enzymatic antioxidant systems cells are equipped with. In particular, thiol-dependent peroxidases peroxiredoxins (PRDXs) have been reported to be major antioxidant enzymes in prokaryotes and eukaryotic cells due to their abundance and high catalytic efficiency for reducing peroxides [4].

In most vertebrates, the PRDX family comprises 6 enzymes (PRDX1–6) divided into three subgroups depending on their catalytic mechanism and the conservation of their catalytic cysteines: typical 2-Cys PRDXs (PRDX1–4), atypical 2-Cys PRDX (PRDX5) and 1-Cys PRDX (PRDX6) [5, 6]. While all PRDX isoforms reduce peroxides via the nucleophilic attack of their peroxidatic cysteine (Cp), leading to the formation of a sulfenic acid (Cp-SOH), the resolution step of the catalytic cycle differs for each subgroup. The oxidized Cp forms a disulfide bond with the resolution cysteine (Cr) of another PRDX subunit for typical 2-Cys PRDXs, or with the Cr of the same enzymatic subunit for atypical 2-cys PRDX5 [7]. For the 1-cys PRDX6, reduction of the Cp sulfenic acid occurs through condensation with an external thiol, generally glutathione in the presence of glutathione-S-transferase (GST) π [8–10].

With regards to other PRDXs, PRDX5 appears to be a particular PRDX whose cytoprotective function is well documented. Indeed, in addition to its different structure and catalytic mechanism, human PRDX5 shows wider substrate specificity, reducing hydrogen peroxide, but also alkyl hydroperoxides and peroxynitrite, and a broad subcellular distribution, localizing to the mitochondria, the cytosol, the peroxisome and, in some situations, to the nucleus [11]. These multiple localizations are a consequence of the existence of two PRDX5 isoforms encoded by a single *PRDX5* gene containing alternative transcription start sites and two in-frame translation initiation sites [12]. The short form of PRDX5 (S-PRDX5) will be found in the cytosol and nucleus, but also in peroxisomes thanks to a weak carboxy-terminal peroxisomal targeting sequence type 1 (PTS1). The long form of PRDX5 (L-PRDX5) contains an amino-terminal mitochondrial targeting sequence (MTS) which is cleaved after mitochondrial import, producing a mature mitochondrial PRDX5 identical to the short form [12–14]. PRDX5 is an evolutionarily conserved enzyme, PRDX5 orthologs existing throughout the animal kingdom, in invertebrates and vertebrates alike [12, 15, 16]. However, although the short form is conserved, recent reports have shown that mitochondrial targeting of PRDX5 is absent in certain mammalian species, including pig and canids [15, 16].

Here we report converging evidence pointing to the loss of the *PRDX5* gene in birds though it is conserved in non-avian amniotes, and lead a thorough discussion concerning the potential biological significance of this absence. Finally, using *in ovo* electroporation, we show that subcellular targeting of human PRDX5 is functional in chick spinal cord and we validate the chick as a novel gain-of-function model for studying PRDX5 function in vivo.

## Methods

### Computer analysis

Human PRDX5 and TRMT112 mRNA (*PRDX5*: NM_012094.4; *TRMT112*: NM_001286082.1), and protein (PRDX5: NP_036226.1; TRMT112: NP_001273011.1) sequences were obtained from National Center for Biotechnology Information (NCBI). Identification of predicted PRDX5 and TRMT112 orthologs in other species was carried out using NCBI Blast, as well as UCSC and International Crocodilian Genomes Working Group (ICGWG) genome browsers [17]. Translation of nucleotide sequences was performed via the ExPaSy translate tool [18]. Multiple sequence alignment was done with Clustal Omega [19], and displayed using GeneDoc with the conserved residue shading mode and similarity groups enabled [20]. TargetP 1.1. [21, 22] and Mitoprot [23] were used for mitochondrial targeting prediction.

### Antibodies

Primary antibodies used in the present study were previously validated and described [24, 25]. References of the antibodies, as well as dilutions used for Western blotting and immunofluorescence analyses are specified in Table 1. Mouse monoclonal antibodies anti-ATP synthase subunit beta (ATPB; 3D5, Abcam) and anti-catalase (CAT; CAT-505, Sigma) were used as markers of mitochondria and peroxisomes, respectively. Secondary antibodies Alexa Fluor 546 Donkey anti-Mouse IgG and Alexa Fluor 633 Donkey anti-Goat IgG were obtained from Invitrogen. The background signal generated by the secondary antibodies was tested and considered negligible.

**Table 1:** Primary antibodies

Antigen	Source	Dilution
PRDX1	Rabbit polyclonal, obtained in Hormonology laboratory of Marloie (Belgium), No. UC232	1:4000 for Western blotting
PRDX2	Rabbit polyclonal, obtained in Hormonology laboratory of Marloie (Belgium), No. UC197	1:5000 for Western blotting
PRDX3	Rabbit polyclonal, obtained in Hormonology laboratory of Marloie (Belgium), No. UC210	1:5000 for Western blotting
PRDX4	Rabbit polyclonal, obtained in Hormonology laboratory of Marloie (Belgium), No. UC194	1:5000 for Western blotting
PRDX5	Rabbit polyclonal, obtained in Hormonology laboratory of Marloie (Belgium), No. G234	1:1500 for immunofluorescence 1:5000 for Western blotting
ATPB	Abcam; catalog No. ab14730, mouse monoclonal (3D5)	1:500 for immunofluorescence
CAT	Sigma; catalog No. C0979, mouse monoclonal (CAT-505)	1:100 for immunofluorescence

### Animal and tissue processing

Chicken tissue was obtained from an adult male red junglefowl (*Gallus gallus*), kindly provided by a farm in Tilly (Belgium). Owner of the animal verbally consented to its use in the present study. Fertilized eggs were obtained from Wyverkens farm (Halle, Belgium). Animal and tissue processing was performed according to protocols described in Pirson and Knoops (2015) and Pirson et al. (2015) [25, 26].

### Western blotting

Western blotting of chicken tissue homogenate proteins and SH-SY5Y (human neuroblastoma) cell lysate proteins, serving as a positive control, was performed as previously described [25, 26].

### Cloning of human L-PRDX5 and S-PRDX5 cDNA

Cloning and purification of human *L-PRDX5* and *S-PRDX5* cDNA were performed as described previously [27]. The human *PRDX5* cDNA was PCR-amplified with forward primer 5′-GGCCGT*GAATTC*GGTATGGGACTAGCTGGC-3′ (*Eco*RI site is in italics) for *L-PRDX5* or 5′-AGAGCC*GAATTC*GCCATGGCCCCAATCAAG-3′ (*Eco*RI site is in italics) for *S-PRDX5* and reverse primer 5′-TAATCT*GCGGCCGC*GCCTCAGAGCTGTGAGAT-3′ (*Not*I site is in italics). After restriction, the sequence was ligated into pCMS-*eGFP* vector (Clontech, Palo Alto, CA, USA) in which *L-PRDX5* or *S-PRDX5* coding sequences are under the control of a CMV promoter.

### *In ovo* electroporation

Treatment of fertilized eggs and *in ovo* electroporation of chick embryos were performed as previously described [26]. Briefly, pCMS-*eGFP* vector (Empty vector), pCMS-*eGFP* vector with human *L-PRDX5* (*L-PRDX5* vector) or *S-PRDX5* (*S-PRDX5* vector) cDNA sequences were injected into the neural tube of Hamilton-Hamburger stage (HH) 12–14, i.e. embryonic day 2–2.5 (E2–2.5), chick embryos at a concentration of 2 μg/μl prior to electroporation. Subsequent processing (cryosectioning and immunolabeling) was carried out 48 h after electroporation, on stage E4–4.5 embryos.

### Immunofluorescence assay

Immunofluorescence assay and image acquisition of chick embryonic spinal cords was performed as previously described [25, 26].

## Results

### Western blotting analysis of PRDXs in chicken tissue homogenates

**Fig. 1:**
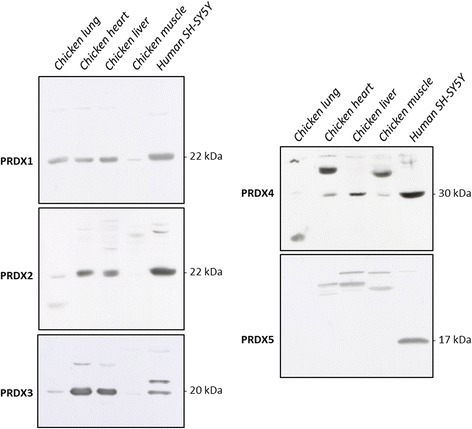
Western blotting analysis of 2-Cys PRDXs in chicken tissue. Proteins from chicken lung, heart, liver, and muscle homogenates and human neuroblastoma SH-SY5Y cell lysates (positive control) were loaded on a SDS-PAGE gel and probed with antibodies directed to 2-Cys PRDXs (see Table 1). Molecular weights (kDa) are indicated

The *PRDX5* gene is conserved throughout evolution and orthologs have been described in a wide array of animal species, ranging from invertebrates to mammals [12, 15, 16]. In chicken (*Gallus gallus*), however, though the existence of PRDX1, PRDX3–4, and PRDX6 isoforms was previously reported, the possible absence of typical 2-Cys PRDX2 and atypical 2-Cys PRDX5 was not discussed [28]. Here we show that absence of PRDX5 in *Gallus gallus* is further supported by Western blotting analyses of chicken lung, heart, liver, and muscle homogenates using antibodies directed towards human 2-Cys PRDX1–5 (Fig. 1). Soluble proteins of human SH-SY5Y cells were used as a positive control.

All antibodies, including anti-PRDX2, yielded a band at the expected molecular weight, with the exception of anti-PRDX5 which produced no signal around the 17 kDa mark. Higher molecular weight bands observed for PRDX3 and PRDX4 were around the 40 kDa and 60 kDa marks and likely correspond to the dimerized form of the enzyme resulting from incomplete reduction of the samples prior to the analysis. The low intensity high molecular weight bands detected for several of the enzymes, including PRDX5, were deemed to be linked to aspecific binding of the antibody.

### Identification of PRDX5 orthologs in amniotes

**Fig. 2:**
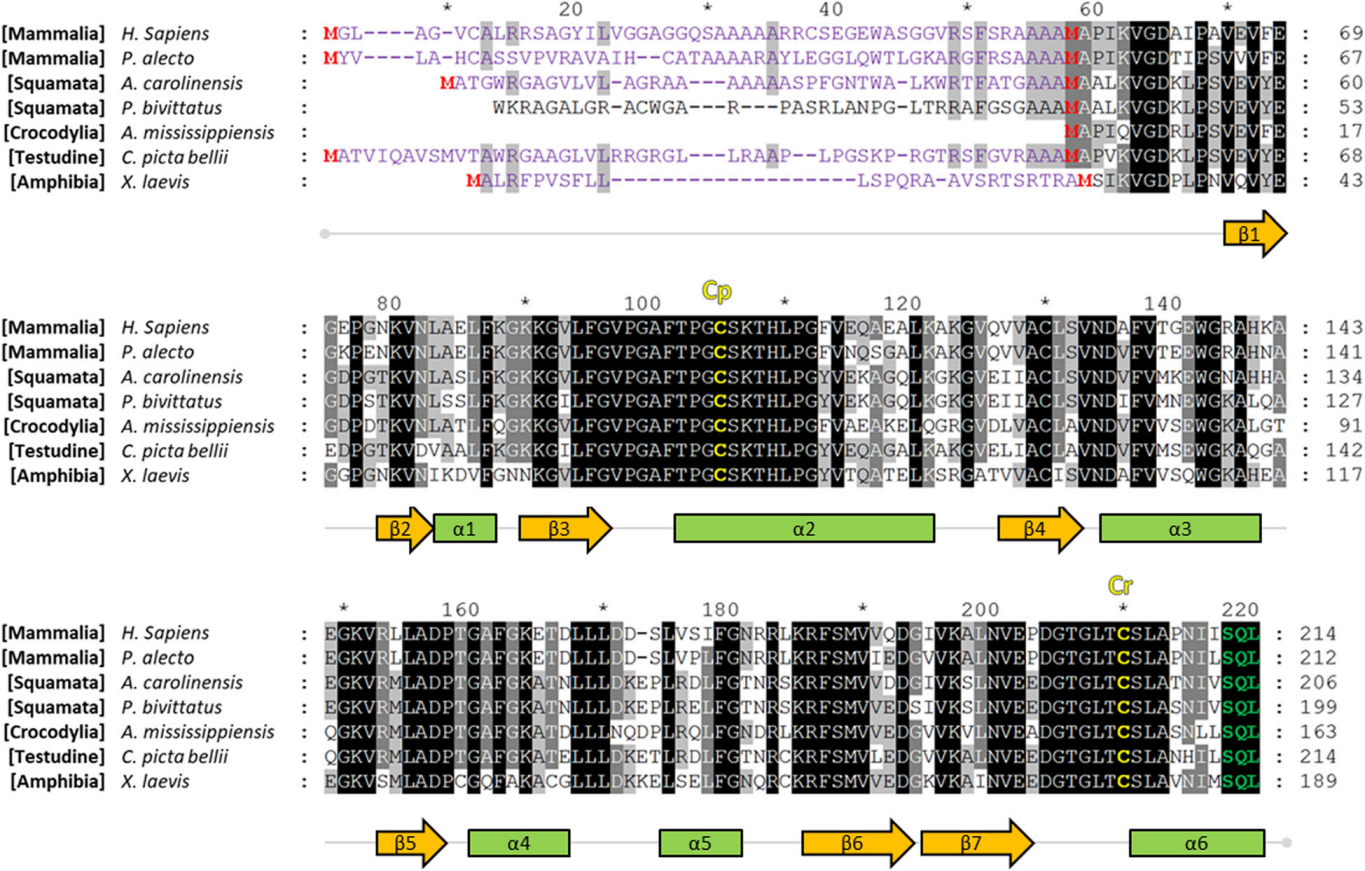
Amino acid sequence alignment of PRDX5 orthologs in amniotes and *Xenopus laevis* amphibian. Multiple sequence alignment was performed with Clustal Omega, and displayed using GeneDoc with the conserved residue shading mode and similarity groups enabled. Letters on a black background represent residues conserved in 100% of the sequences at the corresponding position; residues shown on a gray background are conserved in > 80% of the sequences; residues shown on a light gray background are conserved in > 60% of the sequences. Mitochondrial targeting sequences (MTS) predicted by Mitoprot indicated in purple, and methionine residues aligning with human PRDX5 start methionines are in red. Conserved catalytic cysteines (peroxidatic cysteine, Cp; resolution cysteine, Cr) and peroxisomal targeting sequence (PTS) are indicated in yellow and green, respectively. Human PRDX5 protein secondary structure illustrated below the alignment is from Declercq et al. (2001) [71]. Accession numbers are as follow: NP_036226.1 (human, *Homo sapiens*); XP_006911206.1 (black flying fox, *Pteropus alecto*); XP_003230134.1 (anole lizard, *Anolis carolinensis*); XP_007434436.1 (Burmese python, *Python bivittatus*); KYO29582.1 (American alligator, *Alligator mississippiensis*); XP_005305567.1 (painted turtle, *Chrysemys picta bellii*); XP_018115535.1 (african clawed toad, *Xenopus laevis*). MTS of *Python bivittatus* was investigated using mRNA sequence XM_007434374.1 and WGS sequence NW_006534524.1. *Alligator mississippiensis* PRDX5 sequence was completed using sequences from the *Alligator mississippiensis* AKHW01000000 genome GBrowse

In order to further evidence the loss of PRDX5 in birds during evolution and determine whether the enzyme is conserved in non-avian amniotes, an *in silico* examination of PRDX5 orthologs was carried out and amino acid sequence alignment was performed (Fig. 2). On June 22nd 2017, reference mRNA and protein sequences for *Homo sapiens* (human) PRDX5 were obtained from NCBI sequence databases. These sequences were used to identify predicted orthologs in bats (*Pteropus alecto*, black flying fox), lizards (*Anolis carolinensis*, anole lizard), turtles (*Chrysemys picta bellii*, painted turtle), snakes (*Python bivittatus*, Burmese python), crocodiles (*Alligator mississippiensis*, American alligator), and amphibians (*Xenopus laevis*, african clawed toad) using the blastx function (search a protein database using a translated nucleotide query) on non-redundant protein sequence database. Conversely, this analysis did not yield sequences of considerable homology in aves. Similarly, tblastn analysis (search a translated nucleotide database using a protein query) of predicted *Anolis carolinensis* (anole lizard) PRDX5 protein sequence on all available NCBI databases containing bird sequences generated no potential candidates for PRDX5.

All identified orthologs displayed conserved peroxidatic cysteine (Cp), resolution cysteine (Cr) residues and surrounding amino acids, as well as the peroxisomal targeting sequence type 1 (PTS1) at the carboxy-terminus (Fig. 2). Mitochondrial targeting sequences (MTS) were identified in *Pteropus alecto*, *Anolis carolinensis*, *Chrysemys picta bellii* and *Xenopus laevis*, but could not be highlighted in *Python bivittatus* or *Alligator mississippiensis* predicted PRDX5 amino acid sequences. To investigate this, analysis of the genomic sequences from which these predicted PRDX5 protein sequences originate was performed. For *Python bivittatus*, a sequence upstream of the start methionine of S-PRDX5 showed strong homology to *Anolis carolinensis* PRDX5 MTS but was not considered as an open reading frame because of indeterminations at the 5′ extremity of the nucleotide sequence. Concerning genomic sequence of *Alligator mississippiensis* containing the *PRDX5* coding sequence, a sequence 5′ to the *S-PRDX5* start codon reveals the presence of an in-phase STOP codon. Similarly, in-phase STOP codons were also present upstream of the *S-PRDX5* in two other crocodilian species, *Crocodylus porosus* and *Gavialis gangeticus* (data not shown).

### TRMT112 gene is conserved in birds

**Fig. 3:**
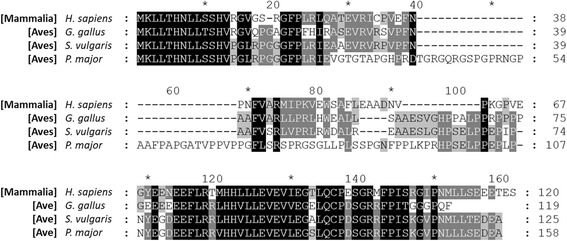
Amino acid sequence alignment of TRMT112 orthologs in human and bird species. Multiple sequence alignment was performed with Clustal Omega, and displayed using GeneDoc with the conserved residue shading mode and similarity groups enabled. Letters on a black background represent residues conserved in 100% of the sequences at the corresponding position; residues shown on a gray background are conserved in > 80% of the sequences; residues shown on a light gray background are conserved in > 60% of the sequences. Accession numbers are as follow: NP_001273011.1 (human, *Homo sapiens*), XP_015130547.1 (red junglefowl, *Gallus gallus*), XP_014749547.1 (common starling, *Sturnus vulgaris*), XP_018861060.1 (great tit, *Parus major*)

The absence of *PRDX5* in available *Gallus gallus* sequences could be linked to rearrangements affecting syntenic gene cluster or due to gaps or errors in genomic sequences available for birds. In order to examine these possibilities, avian orthologs of *TRMT112*, a gene located closely to the 5′ extremity of *L-PRDX5* gene in mammals [15], were identified (Fig. 3). To this end, on June 22nd 2017, human *TRMT112* mRNA and protein sequences were used to identify orthologs in birds by blastp (search a protein database using a protein query) on non-redundant protein databases for aves and tblastn on expressed tag sequence (EST) databases for aves. Predicted *TRMT112* orthologous sequences were identified for several bird species including *Gallus gallus* (red junglefowl), *Sturnus vulgaris* (common starling), and *Parus major* (great tit). Inspection of genomic regions adjacent to *TRMT112* gene revealed no trace of the *PRDX5* gene in these species (data not shown).

### Overexpression of human L-PRDX5 and S-PRDX5 in *Gallus gallus*

**Fig. 4:**
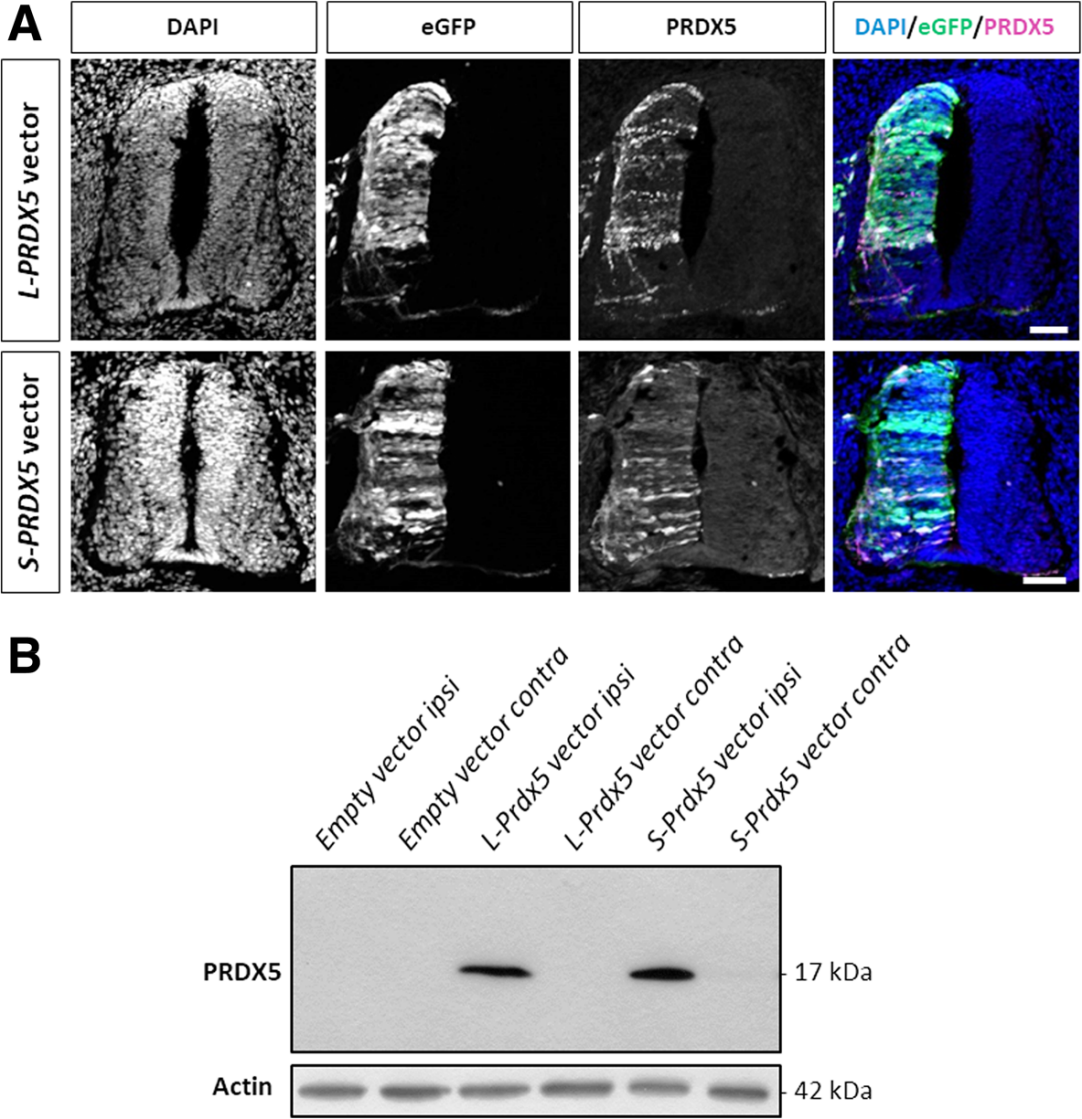
Expression of *L-PRDX5* and *S-PRDX5* by *in ovo* electroporation. **a**. Immunofluorescence detection of PRDX5 in E4.5 chick embryo spinal cords electroporated with L-PRDX5 and S-PRDX5 vectors. eGFP signal serves as control of effective plasmid electroporation. Nuclear DAPI staining is used to visualize cells in the tissues. Scale bars = 50 μm. **b**. Western blotting analysis of the contralateral (non-electroporated) and ipsilateral (electroporated) sides of dissected spinal cords of E4.5 chick embryos electroporated with empty vector, L-PRDX5 vector, or S-PRDX5 vector

**Fig. 5:**
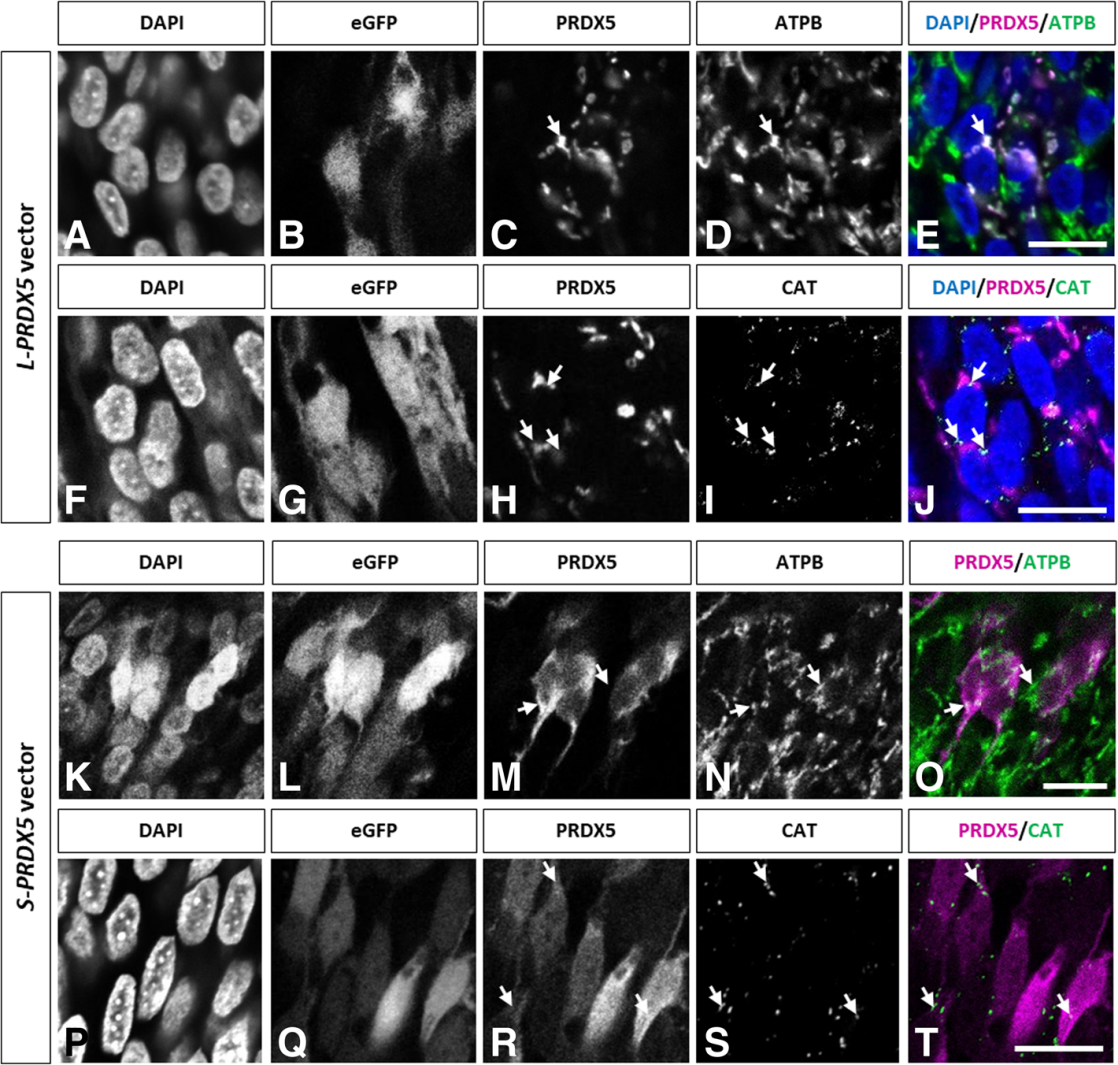
Subcellular localization of L-PRDX5 and S-PRDX5 in chick spinal cord electroporated with *L-PRDX5* and *S-PRDX5* vectors. Co-immunodetection of L-PRDX5 (**a-j**) and S-PRDX5 (**k-t**) with mitochondrial marker ATPB and peroxisomal marker CAT in cryosections of embryonic chick spinal cord. eGFP signal serves as control of effective plasmid electroporation. DAPI staining is used to visualize cell nucleus. Scale bars = 10 μm

Though cellular models such as cancerous cell lines are a useful tool to study protein function, these models are generally oversimplifications of actual biological systems as they do not account for the complex cellular composition, interactions, and environment that exist in vivo and may fail to reproduce key physiological processes. For this reason, the use of in vivo models is critical to confirm data obtained in cellular or in vitro models, and obtain a clearer view of protein function in the context of a live tissue or organism. Although in vivo models, including knockout and transgenic mice models, exist for all typical 2-cys PRDXs (Prdxs1–4) and 1-cys PRDX6, and have offered precious clues to understanding the function of these enzymes, so far no such models have been published for PRDX5 [29–31]. Thanks to technical advances in chick embryo genetic manipulation, the absence of *PRDX5* gene in birds opens the door to the development of an interesting gain-of-function model to study both long and short forms of PRDX5. To this end, injection of DNA constructs containing cDNA sequences coding for human L-PRDX5  and S-PRDX5 into the neural tube and *in ovo* electroporation were performed on stage HH12–14 chick embryos (E2-E2.5). Forty-eight hours after electroporation, immunofluorescence assay using polyclonal antibody directed to human PRDX5 revealed intense signal in the ipsilateral (electroporated) as opposed to the contralateral (non-electroporated) side of the spinal cord in embryos electroporated with both *L-PRDX5* vector and *S-PRDX5* vector (Fig. 4a-b). Concordantly with these results, electroporation of the empty vector did not produce any immunostaining associated to PRDX5 (Fig. 4b). In order to determine whether subcellular targeting of human L-PRDX5 and S-PRDX5 is conserved in *Gallus gallus* and further validate our gain-of-function model, colocalization analysis of overexpressed PRDX5 and subcellular markers were performed on electroporated chick embryos 48 h after electroporation (E4.5) (Fig. 5). Electroporation of *L-PRDX5* vector yielded a punctuated cytoplasmic staining for PRDX5 that colocalized with mitochondrial marker ATP synthase subunit beta (ATPB) (Fig. 5a-e). No overlap of L-Prdx5 and peroxisomal catalase (CAT) signals was observed (Fig. 5f-j). Chick embryos electroporated with *S-PRDX5* vector showed homogenous PRDX5 immunoreactivity in cytoplasm, but also in nucleus. Staining colocalized with peroxisomal marker CAT, however, accumulation in the organelle was not obvious (Fig. 5p-t). Though the signals associated to S-PRDX5 and mitochondrial marker ATPB might overlap to a certain extent, this was not systematic in electroporated cells and might be artefactual (Fig. 5k-o).

## Discussion

### PRDX5 is lost in aves but conserved in non-avian amniotes

The major observation made in this work is the loss of the *PRDX5* gene in birds during evolution, despite the fact that orthologous PRDX5 enzymes are described or predicted in non-avian amniotes, including reptiles, such as the anole lizard, the painted turtle, the Burmese python, and the American alligator, as well as human and flying mammal *Pteropus Alecto*.

This conclusion is supported by several converging lines of evidence. First, Western blotting of chick tissue homogenates with antibodies directed against 2-Cys PRDXs highlighted the presence of bands at expected molecular weights for all PRDXs except PRDX5. This comes as a surprise for the antibody directed to PRDX2 as this enzyme is also considered absent in chicken [28]. However, high degree of homology between human PRDX1–2 used for generation of the antibodies might have led to aspecific binding of the anti-PRDX2 antibody with chicken PRDX1. It is of note that failure of the anti-PRDX5 antibody to recognize an orthologous PRDX5 in chick tissue seems unlikely considering that it is a polyclonal antibody and that PRDX5 orthologs share important sequence homology (> 69% identity in the amino acid sequence between the human protein used to generate the antibody and the PRDX5 of species considered in Fig. 2). Indeed, anti-PRDX5 antibody used in this work has been shown to detect PRDX5 in several other species including mouse, rat, pig, dog, and African clawed toad [15, 16, 24, 32].

To further support the hypothesis that the *PRDX5* gene is absent in chicken, and birds in general, and determine whether the enzyme is present in other amniotes, an *in silico* search was carried out to identify PRDX5 orthologs in different amniote species. Blast screening of available bird sequences in NCBI databases did not enable us to highlight the existence of an avian *PRDX5* gene. Conversely, PRDX5 orthologs were identified in several sauropsida species, including anole lizard, painted turtle, and American alligator, suggesting disappearance of *PRDX5* occurred after the bird-lizard split about 254 million years ago (Triassic period) [33]. Determination of a more precise timeframe would require sequence data of now extinct species.

A major criticism of the identification of genes in this way is the dependence of the methodology on complete and reliable genomic sequences and expressed tag sequence (EST) libraries. Moreover, bird genomes are known to be smaller compared to that of other amniotes [34]. This reduction in genome size has been associated to loss of non-coding DNA but also of protein coding genes. Indeed, chicken genome comprises about 15,500 genes compared to approximately 20,800 in human and 18,600 in the anole lizard [35]. Furthermore, the important loss of protein coding genes during evolution of the avian genome seems linked to high rates of chromosomal rearrangements affecting syntenic blocks of genes [34–36]. For these reasons, identification of *TRMT112* (previously named *HSPC152*), a gene closely located to the *PRDX5* gene, was carried out. No *TRMT112* ortholog could initially be identified from *Gallus gallus* genomic sequences, suggesting the *TRMT112-PRDX5* locus could be subject to gaps, errors, or indeterminations. However, genomic sequences corresponding to *TRMT112* were identified in the common starling and the great tit, and mRNAs corresponding to *TRMT112* were highlighted in chicken suggesting that genes located in this locus can effectively be identified in aves. Moreover, analysis of the sequences situated in the vicinity of the *TRMT112* gene in these species further supports that birds are indeed devoid of the *PRDX5* gene.

The absence of PRDX5 in birds suggests that this enzyme is not essential to these species and the evolutionary success encountered by the avian class does not seem to have been affected by this loss. This highlights questions concerning redox regulation and oxidative stress response in aves. Interestingly, the striking longevity displayed by birds compared to mammals of equivalent mass has, in part, been attributed to increased resistance to oxidative stress or reduced oxidative damages [37, 38]. Indeed, birds have been shown to be more resistant to lipid peroxidation than rodents due to the different composition of their biological membranes [39–41]. Ogburn et al. (1998) also demonstrated that, in oxidative stress conditions, bird epithelial kidney cells exhibited less DNA damage compared to that of rodents [41]. Furthermore, bird mitochondria appear to produce less hydrogen peroxide than rodent mitochondria [42–44]. Due to the physiological adaptations associated to the evolution of flight, bats display similarities to birds with regards to oxidative stress, a fact which has been linked to their remarkable longevity [38]. Indeed, contrasting with their high metabolic rates, both bats and birds exhibit reduced reactive species production in basal and stimulated conditions, as well as increased resistance to oxidative damage compared to non-flying mammals [38, 45, 46]. Along these lines, flight driven aerobicity has been proposed to act as an oxidative sink wherein mitochondria, due to their energetic metabolism and elevated antioxidant defences, would actually be consumers rather than producers of reactive species [37]. Due to these adaptations, it could be hypothesized that PRDX5 may no longer have been essential to antioxidant defence in these species. Conversely to birds, *PRDX5* was readily identified in black flying fox suggesting that the physiological characteristics associated to both birds and bats are not sufficient to explain the disappearance of *PRDX5*. However, evolutionary divergence of birds largely predates that of bats and physiological changes leading to reduced oxidative damages might be less important in bats than in birds [47]. Moreover these species might have followed different evolutionary paths in order to cope with the adaptations.

Another explanation to the loss of PRDX5 could reside in functional redundancy with other antioxidant enzymes. Indeed several other peroxide reductases, including several PRDXs, catalase, or members of glutathione peroxidase family, are conserved in birds [28, 48–51]. Interestingly, lower activity of several antioxidant enzymes and molecules has been reported in bird compared to rodent brain, lung, and liver suggesting that birds might not need the same degree of antioxidant defence [52–54]. Therefore, the disappearance of PRDX5 in birds might be the consequence of functional redundancy with regards to other antioxidant enzymes, as well as reduced necessity for birds to protect themselves from oxidative damage.

It is worth mentioning that, in addition to its well-studied cytoprotective function, other roles have been attributed to PRDX5, notably in immune and inflammatory processes. Indeed, PRDX5 has been reported to be overexpressed in immunostimulated macrophages [55, 56], while extracellular PRDX5 fragments were shown to induce macrophage infiltration via toll-like receptor activation [57]. Moreover, PRDX5 has been negatively linked to pro-inflammatory JNK signaling leading to decreased resistance to infection in drosophila [58]. Coincidentally, the immune system of birds appears to be quite different to that of mammals. Kaiser et al. (2005) reported the production of different cytokines and chemokines during immune response in chicken compared to other vertebrates suggesting distinct immune mechanisms in these species [59]. Moreover, birds display reduced immune gene repertoires compared to mammals [60]. Therefore, the function of PRDX5 in mammalian immune response may no longer have been required in the context of avian immunity and thus the gene ceased undergoing selection.

### Crocodilian PRDX5 lacks a functional mitochondrial targeting sequence

An anecdotal finding of this work is the abolition of the MTS of Alligator PRDX5. Indeed, analysis of the sequence located upstream of the start methionine of S-PRDX5 highlighted the presence of a translation STOP in the MTS. PRDX5 mitochondrial targeting is conserved in human, black flying fox, anole lizard, Burmese python, African clawed toad, as well as numerous other vertebrate and invertebrate species [13, 16, 61–67]. However, disruption of mitochondrial targeting of PRDX5 is thought to have occurred on at least two separate occasions during mammalian evolution, namely in pig and canids. In addition, expression of the mitochondrial isoform of PRDX5 in canine MDCK cells had deleterious consequences to the cell upon peroxide exposure [15, 16]. It is possible that L-PRDX5 began exhibiting a similar cytotoxic effect in the crocodile lineage and that mitochondrial targeting was therefore counter-selected.

Although functional MTS could not be identified in Burmese python due to indeterminations in the genomic sequence, the partial sequence obtained showed strong homology with the MTS of the anole lizard. This suggests that mitochondrial targeting of PRDX5 is indeed conserved in snakes as loss of MTS functionality would likely have led to numerous mutations in this sequence should it no longer be submitted to selective pressure. It is important to note that this result is based solely on currently available sequence data and remains speculative until the mitochondrial targeting of PRDX5 in snakes and crocodiles is verified experimentally. Also, it is worth mentioning that the PTS1 is conserved in all PRDX5 sequences analyzed in this study.

### A new in vivo PRDX5 gain-of-function model

In addition to being interesting from an evolutionary standpoint, the absence of PRDX5 in birds also offers the opportunity to develop several gain-of-function models to study PRDX5. Though a cell culture approach through commonly used DT40 chicken B cell line could be envisioned, over the last decade, *in ovo* electroporation has been widely used for gain- or loss-of-function studies during chick embryonic development [26, 68]. This is especially interesting in light of recent reports of a particular expression pattern of PRDX5 in the mouse embryonic spinal cord [25].

In the present study, with the aim of developing the *in ovo* electroporation of PRDX5 model, DNA constructs coding for long and short isoforms of human PRDX5 were electroporated into the embryonic chick spinal cord. Both L-PRDX5 and S-PRDX5 were successfully overexpressed as shown by the strong immunostaining observed on the ipsilateral (electroporated) side of the spinal cord as opposed to the contralateral (non-electroporated) side. Moreover, localization of these forms of the enzyme was coherent with expected subcellular targeting. Indeed, *L-PRDX5* vector produced signal that specifically colocalized with mitochondrial marker ATPB while the *S-PRDX5* vector generated staining in the cytosol, nucleus, and peroxisomes as highlighted through colocalization with catalase. Though our results suggest that PRDX5 is effectively relocated to peroxisomes, lack of stronger signal in peroxisomes compared to cytoplasm may indicate that peroxisomal targeting of PRDX5 is insufficient to lead to accumulation of the enzyme in this organelle. Interestingly, study of the expression pattern of PRDX5 during mouse spinal cord development could not highlight colocalization of PRDX5 with catalase suggesting that peroxisomal targeting of PRDX5 might be weak at embryonic stages [25]. Though the slight overlap of signals associated to S-PRDX5 and ATPB might be an artefactual result, cytosolic S-PRDX5 signal being hard to distinguish from the complex three-dimensional structures formed by mitochondria, it might also be the result of leaking of the overexpressed S-PRDX5 into the mitochondria. Unexpected entry of cytosolic proteins into the mitochondria has previously been reported, notably for antioxidant enzyme SOD1 in transgenic mice overexpressing the protein [69]. Along these lines, mitochondria have been shown to act as guardians of cytosolic proteostasis (protein homeostasis) via the accumulation of overabundant proteins [70].

The model we propose here offers a unique and powerful tool to study function of PRDX5 in vivo and could yield valuable insight into the function of this enigmatic enzyme. For instance, this model could serve to evaluate the effect of L-PRDX5 and S-PRDX5 gain-of-function on biological processes taking place in the embryonic spinal cord, such as neurogenesis, differentiation, and migration of neuronal and glial cellular subtypes, or motor neuronal programmed cell death. In addition, targeting of specific cellular subtypes using gene promoters could be used to study the function of the enzyme in the context of a given cell type. For example, targeting of microglial cells would provide an opportunity to test hypotheses associated to the immune and inflammation-related functions of PRDX5. Furthermore, more general questions could also be approached, such as the degree of cytoprotection conveyed by this enzyme in live tissue subjected to oxidant or inflammatory stressors such as H_2_O_2_, organic peroxides, peroxynitrite, or LPS. Similarly, the impact of PRDX5 on redox-dependent signaling pathways would also be a question worthy of further investigation.

## Conclusion

In this study, we report that though it is conserved throughout animal evolution, the *PRDX5* gene appears to have been lost in birds. Identification of PRDX5 orthologs in non-avian amniotes shows disappearance of PRDX5 is specific to aves and enables us to date this genetic event to after the divergence of birds, 245 million years ago. Biological significance of loss of PRDX5 in birds remains elusive, but physiological and immunological differences in this class could be explanatory factors. Finally, using *in ovo* electroporation of long and short forms of human PRDX5, we report that subcellular localization mechanisms are conserved and highlight this technique as a valuable gain-of-function model for studying PRDX5 in vivo.

## Abbreviations

ATPB: ATP synthase subunit beta
CAT: Catalase
E: Embryonic day
eGFP: Enhanced green fluorescent protein
HH: Hamilton-Hamburger stage
L-PRDX5: Long form of peroxiredoxin-5
MTS: Mitochondrial targeting sequence
PCD: Programmed cell death
PRDX: Peroxiredoxin
PTS: Peroxisomal targeting sequence
RNS: Reactive nitrogen species
ROS: Reactive oxygen species
S-PRDX5: Short form of peroxiredoxin-5
